# Supplementary material for: Metagenomic Evaluation of Bacterial and Fungal Assemblages Enriched within Diffusion Chambers and Microbial Traps Containing Uraniferous Soils
Source: Microorganisms. 2019 Sep 6;7(9):324. doi: 10.3390/microorganisms7090324 (PMC6780890; doi:10.3390/microorganisms7090324)
Supplement: Supplementary file 1 [file microorganisms-07-00324-s001.zip › Supplementary Information.docx]

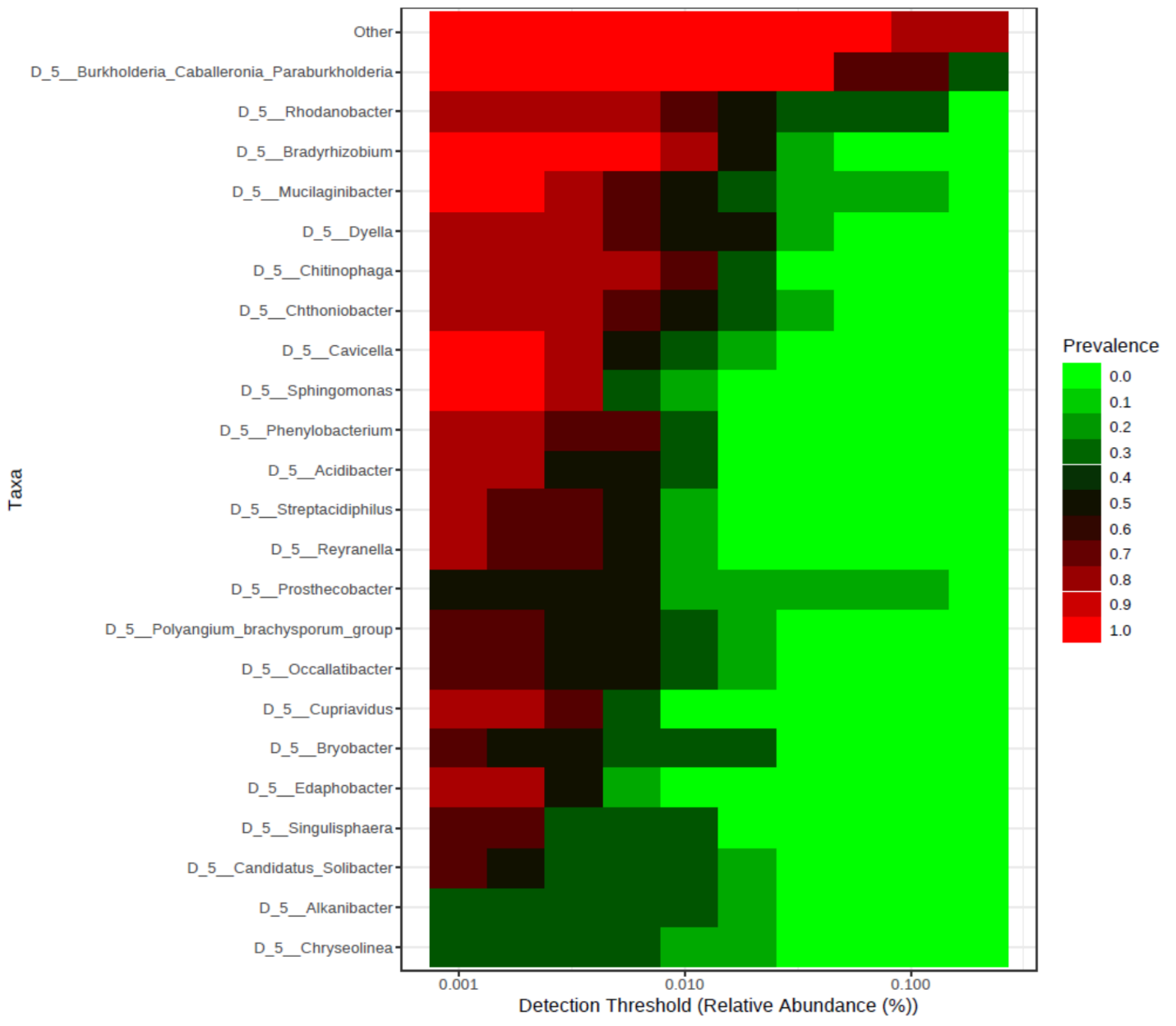


**SI Figure S1.** Shown are the bacterial ‘core’ microbiome at the genus level identified using 16S metagenomic libraries from the DC/MT gels.


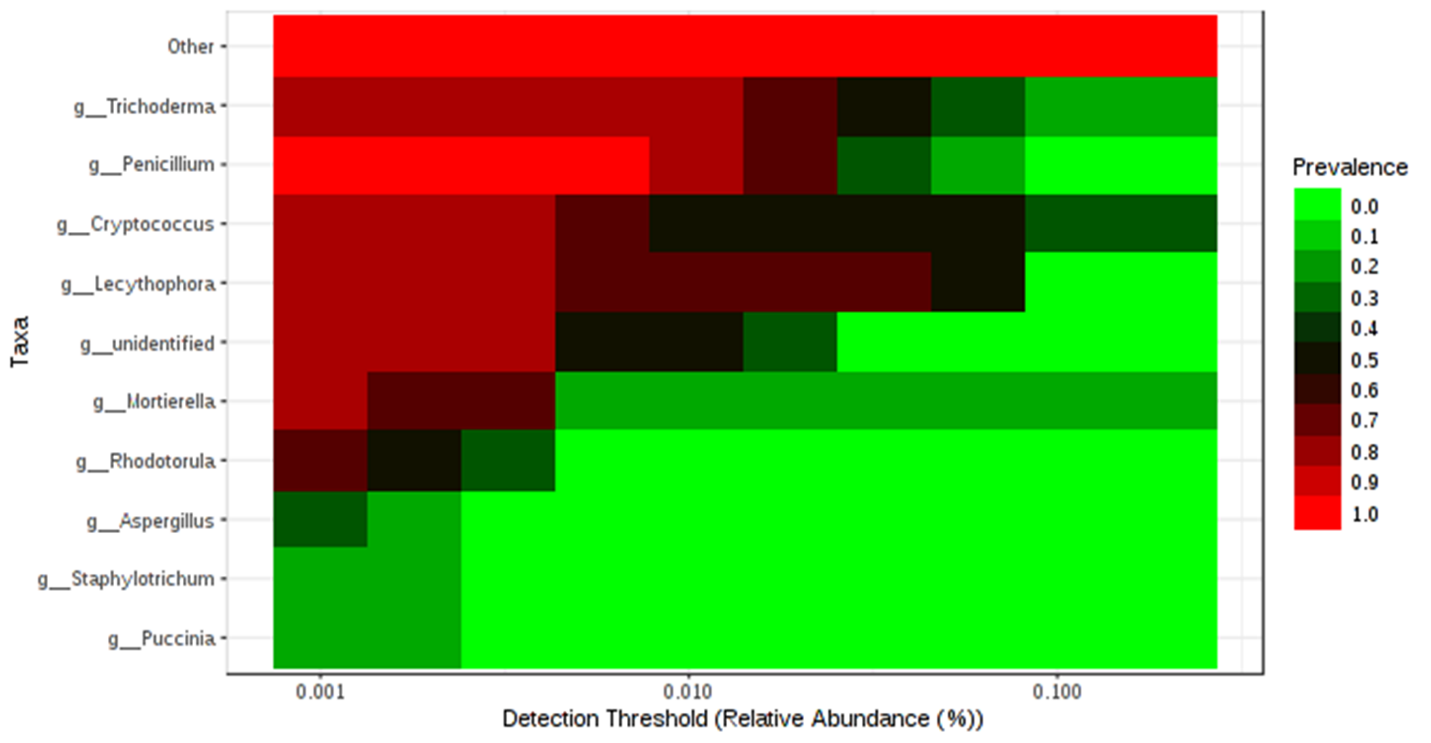


**SI Figure S2.** Shown are the fungal ‘core’ microbiome at the genus level identified using ITS metagenomic libraries from the DC/MT gels.
