# Supplementary figures and images for: Metagenomic Evaluation of Bacterial and Fungal Assemblages Enriched within Diffusion Chambers and Microbial Traps Containing Uraniferous Soils
Source: Microorganisms. 2019 Sep 6;7(9):324. doi: 10.3390/microorganisms7090324 (PMC6780890; doi:10.3390/microorganisms7090324)

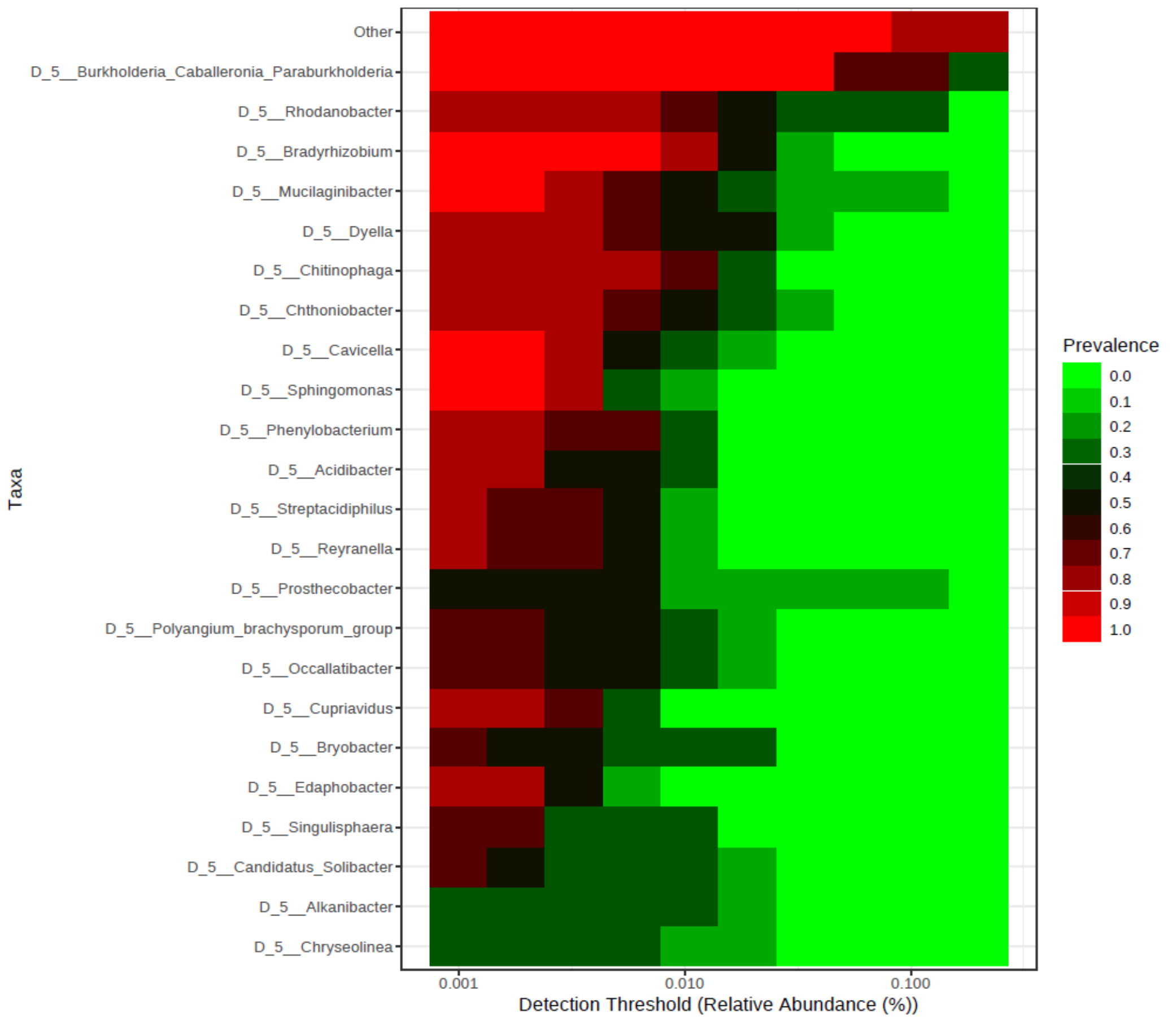

Supplement: Supplementary file 1 [file microorganisms-07-00324-s001.zip › SI. Figure S1 Core Microbiome Bacteria.tif]

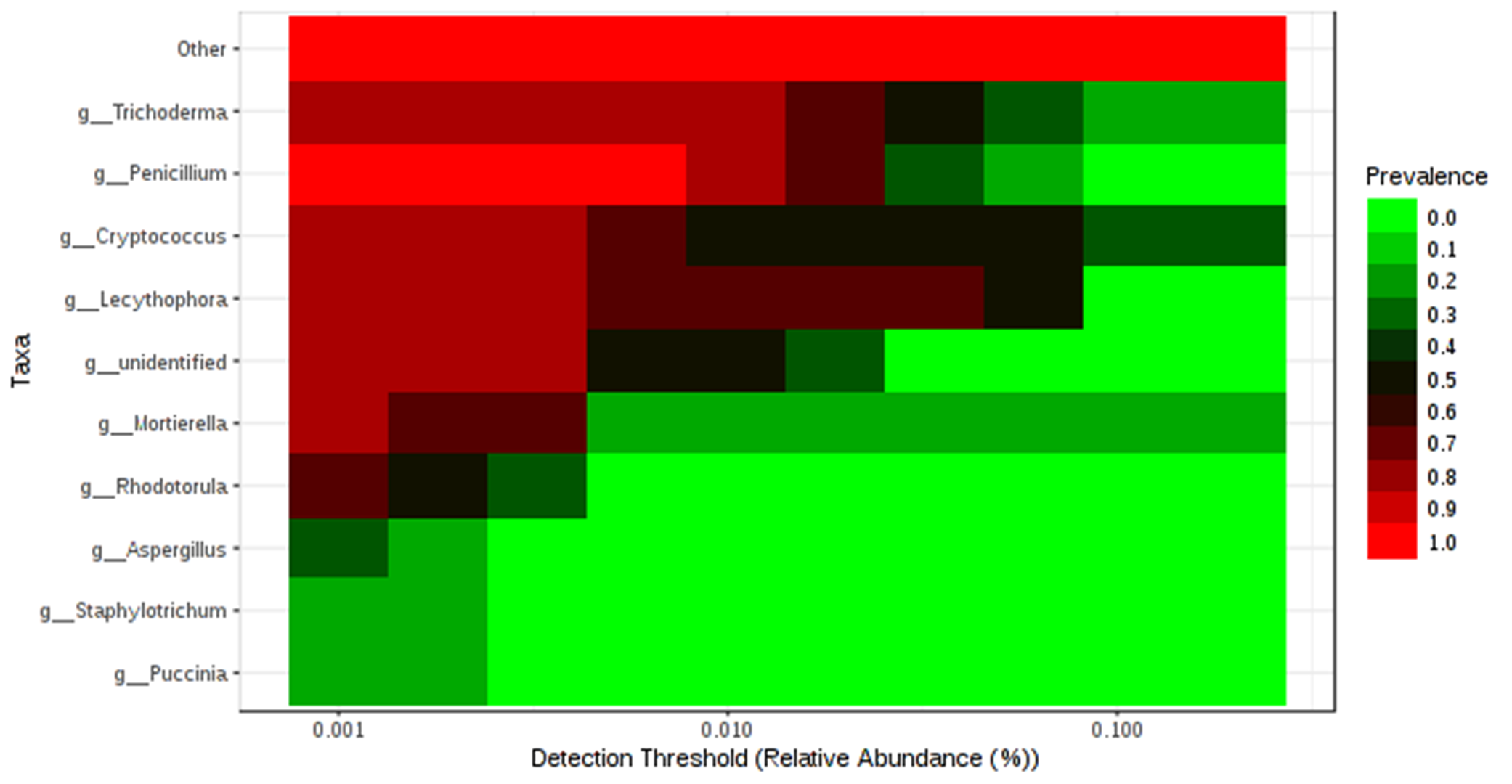

Supplement: Supplementary file 1 [file microorganisms-07-00324-s001.zip › SI. Figure S2 Core Microbiome Fungi.tif]
